# Research on the Power Generation Performance of Solid–Liquid Triboelectric Nanogenerator Based on Surface Microstructure Modification

**DOI:** 10.3390/nano15110872

**Published:** 2025-06-05

**Authors:** Wei Wang, Ge Chen, Jin Yan, Gaoyong Zhang, Zihao Weng, Xianzhang Wang, Hongchen Pang, Lijun Wang, Dapeng Zhang

**Affiliations:** 1 College of Naval Architecture and Shipping, Guangdong Ocean University, Zhanjiang 524088, China; 2 Guangdong Provincial Key Laboratory of Intelligent Equipment for South China Sea Marine Ranching, Guangdong Ocean University, Zhanjiang 524088, China; 3 College of Mechanical Engineering, Guangdong Ocean University, Zhanjiang 524088, China

**Keywords:** SAS-TENG@CIP, material doping, surface modification, percolation theories, electrostatic breakdown (EB)

## Abstract

Since 2015, research on liquid–solid triboelectric nanogenerators (L-S TENGs) has shown steady growth, with the primary focus on application domains such as engineering, physics, materials science, and chemistry. These applications have underscored the significant attention L-S TENGs have garnered in areas like human–nature interaction, energy harvesting, data sensing, and enhancing living conditions. Presently, doping composite dielectric materials and surface modification techniques are the predominant methods for improving the power generation capacity of TENGs, particularly L-S TENGs. However, studies exploring the combined effects of these two approaches to enhance the power generation capacity of TENGs remain relatively scarce. Following a review of existing literature on the use of composite material doping and surface modification to improve the power generation performance of L-S TENGs, this paper proposes an experimental framework termed “self-assembled surface TENG@carbonyl iron particle doping (SAS-TENG@CIP)” to investigate the integrated power generation effects of L-S TENGs when combining these two methods. Research cases and data results indicate that, for TENGs exhibiting capacitor-like properties, the enhancement of power generation performance through composite material doping and superhydrophobic surface modification is not limitless. Each process possesses its own inherent threshold. When these thresholds are surpassed, the percolation of current induced by material doping and electrostatic breakdown (EB) triggered by surface modification can lead to a notable decline in the power output capacity of L-S TENGs. Consequently, in practical applications moving forward, fully realizing the synergistic potential of these methods necessitates a profound understanding of the underlying scientific mechanisms. The conclusions and insights presented in this paper may facilitate their complex integration and contribute to enhancing power generation efficiency in future research.

## 1. Introduction

In 2019, Z. L. Wang and his team introduced “Wang’s Transition” model [1], which elucidates the electron transfer mechanisms between materials in L-S TENGs and describes the formation of a double electric layer at the liquid–solid interface. According to the research findings of Wang’s team, the power generation mechanism of L-S TENGs is analogous to that, both of which operate based on the triboelectric effect. Specifically, mechanical energy is converted into electrical energy through the interaction between a liquid and a solid surface. This contact generates charges on the solid surface and establishes an electric double layer (EDL), thereby facilitating charge accumulation [2,3]. So far, many experimental cases have demonstrated that mechanical energy from liquid–solid interactions can be converted into electrical energy via contact electrification and electrostatic induction. Improved L-S TENGs have been employed to generate triboelectric charges from the interaction of liquids (e.g., water) with solid surfaces, which are then transformed into electrical energy for output [4].

**Figure 1:**
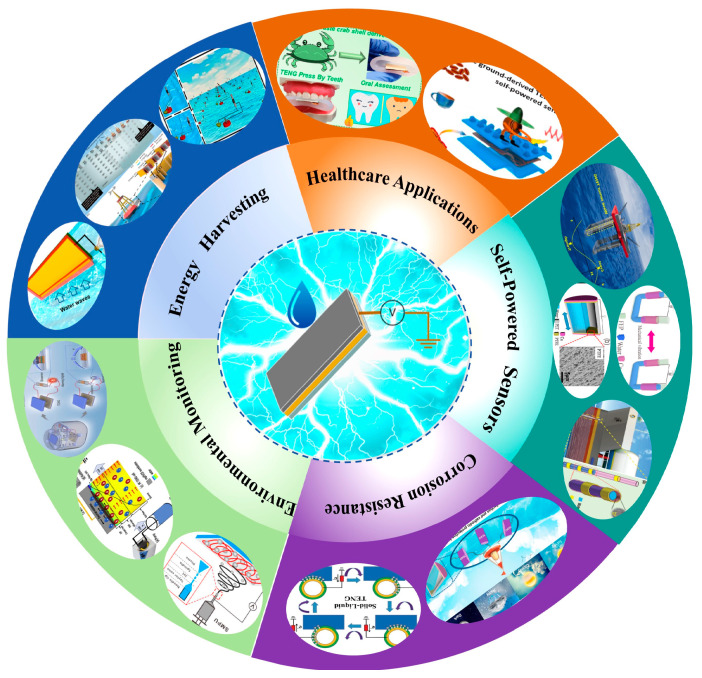
Schematic diagram of L-S TENGs for a wide range of applications.

L-S TENGs harness water energy, which is both abundant and renewable. This characteristic renders liquid–solid triboelectric nanogenerators particularly advantageous due to their lightweight nature, cost-effectiveness, and high energy harvesting efficiency. Consequently, solid–liquid triboelectric nanogenerators have found widespread application in several key areas, as shown in Figure 1 [5,6,7].

In the field of energy harvesting, L-S TENGs efficiently harvest energy from diverse water sources, such as raindrops, rivers, and oceans. They are especially effective for capturing blue energy from oceans, providing a sustainable and eco-friendly solution for powering offshore systems. In the domain of wave and ocean energy harvesting applications, the L-S TENG is capable of harnessing energy from waves, tides, and currents. For instance, Wu et al. (2020) developed a buoy-like L-S TENG aimed at large-scale blue energy collection, showcasing its potential to power marine systems and recharge capacitors effectively [8]. Wei et al. (2021) created a straightforward open L-S TENG (DB-TENG), specifically designed for energy harvesting in marine environments; this system exhibited stable operation under conditions of high humidity and elevated salt concentration [9]. Furthermore, Liu et al. (2020) engineered a comprehensive TENG network comprised of thousands of units for extensive blue energy collection purposes; this configuration achieved a peak voltage of 34 V alongside a transferred charge measurement of 25 nC [10]. In terms of the capability of droplet-based liquid–solid triboelectric nanogenerators (L-S TENGs) to harvest energy from falling raindrops, Xu et al. (2020) further advanced this field by developing a droplet-based L-S TENG with high instantaneous power density, which effectively converted kinetic energy from raindrops into electrical energy [11]. Additionally, Cui et al. (2020) introduced a large-scale droplet L-S TENG utilizing layered double hydroxides (LDH) as the triboelectric layer, showcasing long-term stability and high efficiency in energy conversion [12].

In the field of self-powered sensors research and development, L-S TENGs have gained significant traction as self-powered sensors across diverse fields, including physics, chemistry, and biomedicine. These devices are capable of converting mechanical energy into electrical energy, rendering them particularly effective for real-time monitoring and detection applications. L-S TENGs can be effectively utilized for real-time liquid level monitoring functions akin to those of liquid level sensors. The water level sensor designed by Xu et al. (2019) showcased exceptional accuracy in measuring water levels—its sensitivity being ten times greater than that of conventional methods [13]. A liquid–solid triboelectric nanogenerator served as a water level sensor for ship draft detection; it revealed a linear relationship between water levels and output voltage (Zhang et al., 2020) [14].

In the field of chemical/biological sensor design and development, the capability of L-S TENG to detect variations in liquid properties—such as ion concentration or pH value—led Li et al. (2022) [15] to create a “self-powered ion concentration sensor”. This innovative device demonstrated a linear correlation between NaCl concentration and output voltage, exhibiting high sensitivity within the range of 0.005 M to 0.1 M. In the same year, Wang et al. (2022) [16] developed another self-powered chemical sensor based on L-S TENG for detecting ethanol and formaldehyde concentrations in water, achieving an impressive detection limit of 0.1 ng/L for formaldehyde. Subsequently, in 2022, Li et al. designed a sweat sensor utilizing L-S TENG technology to monitor the NaCl concentration in perspiration, thereby providing real-time feedback regarding physical condition during exercise [17]. A high-entropy ceramic-based L-S TENG was used as a biosensor to detect bacterial concentration, offering higher sensitivity and stability compared with traditional sensors (Wang et al., 2021) [16].

### 1.1. Environmental Monitoring

In the domain of engineering applications for environmental monitoring, such as marine environment monitoring, inorganic non-biological components in water—including pH value and ion concentration, as well as organic biological components like Gram-negative bacteria (Gnb) (sulfate-reducing bacteria)—are the primary agents inducing microbiologically influenced corrosion (MIC) in marine environments, resulting in significant challenges for marine engineering. The integration of L-S TENG technology in the development and design of sensors for environmental applications can provide a precise solution to these issues. For instance, Xiong et al. (2019) designed a self-powered water temperature sensor that integrates a superhydrophobic TENG with a water quality sensor, achieving real-time monitoring of parameters such as water temperature, pH value, and ion concentration with high precision [17]. Furthermore, Zhou et al. (2022) developed a microbial sensor system based on a triboelectric nanogenerator (TENG), which can instantly detect Gram-negative bacteria in solutions, such as sulfate-reducing bacteria [18].

### 1.2. Healthcare Applications

Within the realm of healthcare applications, L-S TENGs have demonstrated potential for integration into wearable devices to monitor physiological signals, including breathing, pulse, and movement. For instance, Li et al. (2021) developed a TENG based on coffee grounds that can monitor breathing and pulse and distinguish between normal breathing patterns and shortness of breath [19]. In a similar vein, Panda et al. (2023) developed a TENG derived from biomaterials, which functions as an autonomous oral health sensor [20]. This sensor is capable of detecting dental defects, such as cracks and misalignment, through the measurement of bite force.

### 1.3. Corrosion Resistance

Liquid–solid triboelectric nanogenerators have demonstrated considerable promise in the field of cathodic protection, particularly in marine environments. These devices have been shown to supply power to metal anti-corrosion systems, thereby ensuring the continuous delivery of external current to prevent metal corrosion. The energy generated by liquid–solid TENGs can induce electrochemical changes, which in turn help to protect materials from corrosion. In 2021, Sun et al. pioneered a novel liquid–solid TENG array combination for self-powered cathodic protection systems in marine environments [21]. Their innovative design utilized polytetrafluoroethylene ultrafiltration membranes as the friction layer and water as the carrier to collect wave energy. In a subsequent study, Wu et al. (2022) designed a hybrid spherical TENG for the cathodic protection of steel [22]. They successfully constructed a hybrid spherical triboelectric nanogenerator (S-TENG) with both solid–solid and solid–liquid contact modes. Their research indicated that the potential drops of stainless steel (304SS) and organic-coated carbon steel (Q235CS) combined with S-TENG were approximately 410 mV and 930 mV, respectively. These findings suggest that S-TENG has the potential to function effectively in marine environments, making it suitable for cathodic protection applications in such settings.

**Figure 2:**
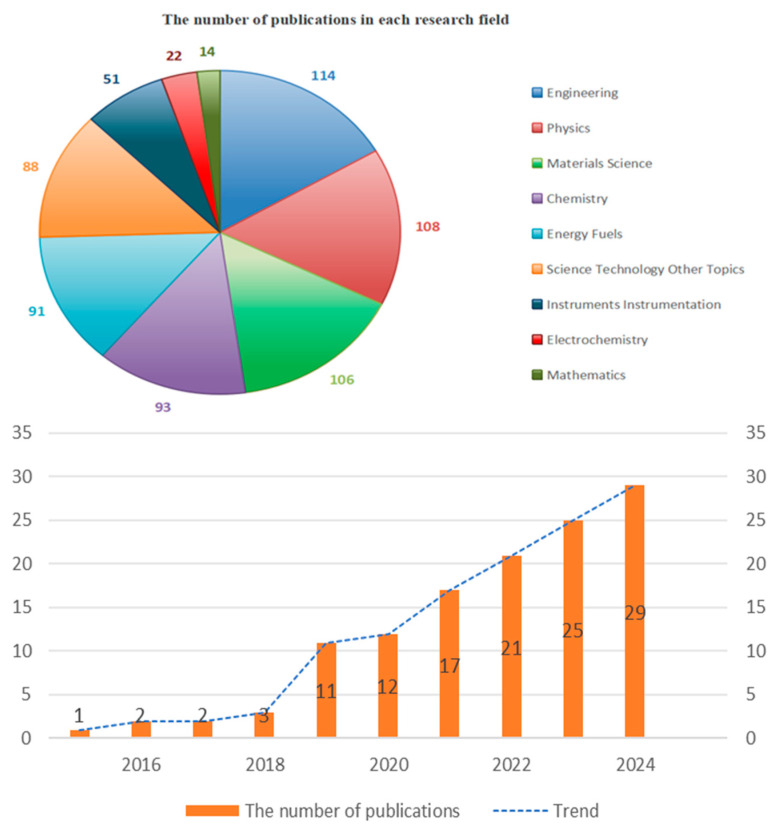
Research fields and publication trends of L-S TENGs over the past 10 years.

As demonstrated in Figure 2, there has been a steady rise in research on L-S TENGs since 2024, with a predominant focus on applied disciplines, such as engineering, physics, materials science, and chemistry. Notably, Jeong Young Park’s team advanced non-contact TENGs by leveraging ferroelectric materials [23], Co-NPC/Ecoflex and MXene/Ecoflex nanocomposites based on metal–organic frameworks [24], as well as siloxane/Ecoflex composites incorporating MoS2 and laser-induced graphene (LIG) as charge-capturing intermediate layers [25]. Shuangxi Nie’s team developed polyvinylidene fluoride@Mxene (Ti_3_C_2_T_x_) composite films with spherical multi-physical network structures via controlled electrospinning for non-contact TENG applications [26]. David Vera Anaya et al. designed non-contact TENG sensors for elderly health monitoring [27]. Additionally, Chuqiao Lyu, Wenbo Ding, and Changsheng Wu introduced a non-contact triboelectric detector (CTD) capable of classifying complex motion patterns in 3D space [28].

The various applications mentioned above underscore the significant attention being directed toward L-S TENGs in areas like human development of nature, energy extraction, data perception, and enhancing living conditions. Over the past decade, numerous research teams have documented various strategies to enhance the power generation performance of L-S TENGs. 

Fundamentally, the TENG can be regarded as a specific type of “special capacitor”. Research on enhancing the power generation performance of L-S TENGs primarily concentrates on two approaches:

(1) The approach involves modifying the dielectric material properties of TENG, such as through the incorporation of additional materials to create a new type of L-S TENG composite dielectric material, thereby enhancing the “special capacitor’s” energy storage capacity.
(2) The approach involves the use of physical or chemical methods to modify the surface properties of TENG dielectric materials, rendering the dielectric surface either hydrophilic or hydrophobic. The overarching objective of these approaches is to enhance the “special capacitor’s” capacity to capture charges in liquid media. It is noteworthy that research on the synergistic application of composite dielectric materials and surface modification techniques to enhance the power generation capacity of triboelectric nanogenerators (TENGs) remains relatively scarce. What specific roles do these two manufacturing processes—composite dielectric materials and surface modification—play in improving the power generation performance of L-S TENGs? Do they exhibit any mutual influence mechanisms, or does one method exert a greater impact than the other? These questions present a series of intriguing and thought-provoking inquiries. To address these questions, this paper reviews and summarizes existing literature concerning the two primary categories—composite materials and surface modification—that aim to improve the power generation performance of L-S TENGs. Subsequently, an experimental framework titled “Self-Assembled Surface TENG@Carbonyl Iron Particles Doping (SAS-TENG@CIP)” is designed to validate the combined effects of composite materials and surface modifications on L-S TENGs. This experimental setup utilizes carbonyl iron nanoparticles along with polydimethylsiloxane (PDMS) as base materials, generating a hydrophobic microstructured surface array through magnetic field manipulation.

In this study, various configurations are tested under the “single-electrode triboelectric nanogenerator” mode: commercial PDMS, carbonyl iron nanoparticle-doped PDMS, SAS-TENG@CIP (spinulose arrays), and SAS-TENG@CIP (T-shaped arrays) serve as inorganic dielectric materials for evaluating their power generation capacities. The results regarding power generation performance attenuation are as follows: SAS-TENG@CIP (spinulose arrays) exhibited the fastest attenuation; followed by SAS-TENG@CIP (T-shaped arrays), showing the second-fastest attenuation; carbonyl iron nanoparticle-doped PDMS demonstrated moderate attenuation; while commercial PDMS displayed no significant attenuation.

This paper employs the latest theories of electrostatic breakdown and current leakage in L-S TENG to elucidate the aforementioned phenomena. Based on this intriguing experimental observation, we provide explanations from two perspectives:

(1) Wang the “TENG” type of “special capacitor”, selecting high-quality composite dielectric materials that minimize current leakage is more critical than merely implementing surface modifications to enhance power generation performance;
(2) The attenuation observed in the SAS-TENG@CIP (spinulose arrays) presented in this study results from a combined effect of both electrostatic breakdown and current leakage mechanisms.

## 2. Strategies for Enhancing the Power Generation of L-S TENG

Since Wang Zhonglin’s team proposed the novel energy technology of triboelectric nanogenerators (TENGs) as self-powered systems in 2013, their application as mechanical and chemical sensors has underscored the significance of material selection and surface engineering in enhancing performance [29,30]. In a 2015 publication by Wang’s team that aimed to define the performance quality factor of TENGs, it was noted that TENGs are characterized by structure-related quality factors and material quality factors associated with the square of surface charge density. In 2023, Yunlong Zi’s team published numerous research papers on the figure of merit (FOM) of triboelectric nanogenerators [31]. In 2025, Haiyang Zou et al. [32] conducted an in-depth analysis of the interplay between the material selection and performance metrics of nanogenerators via a comprehensive literature review. Notably, Jin-Seong Park, Jin Suk Myung, Sang-Jin Lee et al. [33], in their study on enhancing energy harvesting performance by integrating nanostructures with plasma-polymerized fluorocarbon films for light and triboelectric charge management, not only introduced a novel calculation method for the FOM of transparent TENGs, but also demonstrated through a tabular summary that the average FOM of their fabricated TENG devices was six times higher than that of conventional transparent TENGs.

### 2.1. Doped Composite Dielectric Materials

**Figure 3:**
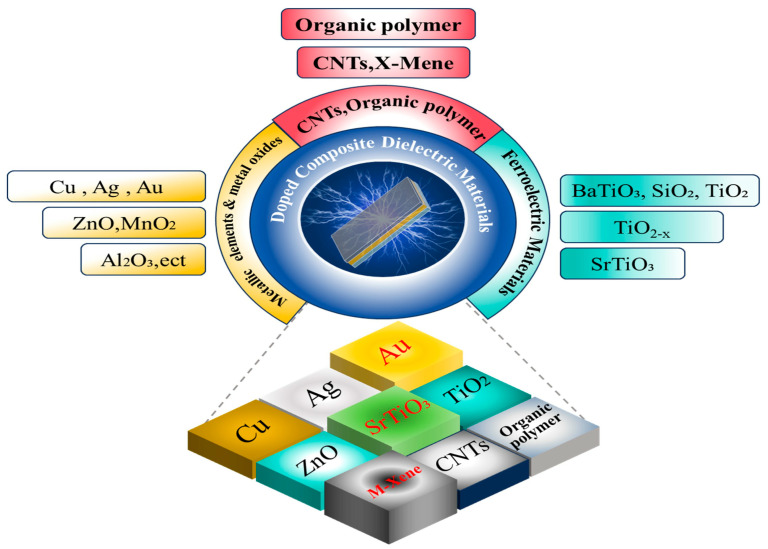
Schematic diagram of various research on doping dielectric materials.

The performance of TENGs is largely contingent upon the dielectric materials employed. Composite-doped dielectric materials can significantly enhance the triboelectric effect by increasing both charge density and surface potential, as these materials influence charge generation, capture, and dissipation. Doped composite dielectric materials have demonstrated considerable improvements in charge density and energy output. Liquid–solid TENGs particularly benefit from composite dielectric materials that facilitate enhanced charge transfer and retention. Utilizing composite dielectric materials—such as polymers doped with metal nanoparticles, metal oxides, ferroelectric substances, carbon nanotubes, organic compounds, or MXenes—can amplify the triboelectric effect and consequently improve energy conversion efficiency (as shown in Figure 3).

In the realm of doping with metallic elements, Zhang et al. [34] developed an innovative triboelectric nanogenerator (TENG) based on a thermoplastic elastomer (TPE) composite fabric doped with nanoparticles (NPs). This TENG operates through a straightforward coating method utilizing NP-doped TPE composite fabric. Upon integrating the TENG with a copper NP-doped TPE film, the resulting composite fabric demonstrated exceptional elastic properties, favorable bending performance, and remarkable flexibility. It achieved a maximum output voltage of 470 V, a current of 24 μA, and power output of 12 mW at an impedance of 3 MΩ. In another study conducted by Chen et al. in 2018, the authors systematically investigated a TENG fabricated using an embedded nanocapacitor structure composed of polytetrafluoroethylene (PTFE) infused with gold nanoparticles (Au-NPs) [35]. Their research characterized the output performance, stability, and durability of the Au-PTFE nanocomposite film-based TENG. Notably, it was observed that, compared with the original PTFE film, there was a significant increase in output current of 70%, while the equivalent surface charge density (ESCD) reached as high as 85 μC/m^2^. Furthermore, Biutty et al. in 2020 prepared a porous polydimethylsiloxane (PDMS) composite material modified with gold nanoparticles aimed at enhancing both compressibility and dielectric constant characteristics of PDMS elastomers [36]. To facilitate uniform deposition of gold nanoparticles on its surface, they synthesized a layer of polydopamine on PDMS.

In recent years, the application of metal oxides as enhanced materials for triboelectric nanogenerator (TENG) power generation has led to the gradual discovery of various remarkable properties associated with transition metal oxides (TMOs). ZnO powder exhibits a relatively high dielectric constant, and appropriate doping can significantly enhance the dielectric constant of room-temperature vulcanized silicone rubber (RTV) films. This enhancement subsequently increases the maximum transfer charge density of TENGs. Mu et al. experimentally demonstrated in 2022 that the open-circuit voltage and short-circuit current of TENGs doped with ZnO were 2.51 times and 2.07 times greater than those of their undoped counterparts, respectively [37]. Bai et al. developed a PNF-TENG in 2020 by incorporating Al_2_O_3_ fillers into an acetate cellulose network, achieving substantial triboelectric charge output [38]. This configuration provided approximately 2.5 mW/cm^2^ electrical performance under an external resistance of 0.8 MΩ. In another study conducted by Zhang et al. in 2022, a porous carbon powder/manganese dioxide (C/MnO_2_) nanocomposite known as CM-TENG was prepared using MnO_2_, resulting in a maximum output voltage of 63 V—approximately 2.1 times that of PDMS-TENG and 1.86 times that of C-TENG—and capable of easily lighting up to 53 LEDs [39]. Moreover, Gao et al. utilized TiO_2_ as a micro-nano capacitor to develop a TENG in 2022; this device featured regular doping with TiO_2_ particles sized at approximately 80 µm and produced around 428.3 nC of sustainable enhanced transfer charge—over threefold compared with that of traditional TENGs [40].

In the domain of utilizing ferroelectric materials as dopants to optimize the charge generation layer in triboelectric nanogenerators (TENGs), Chiang, C.K. et al. introduced a high-dielectric-constant polymer composite material system as early as 2002 [41]. This innovative composite material comprises a high-dielectric-constant cyanate ester resin polymer, which is filled with ferroelectric ceramic BaTiO_3_ powder. In 2016, Jie Chen et al. [42] employed SiO_2_, TiO_2_, BaTiO_3_, and SrTiO_3_ to enhance the relative dielectric constant of polydimethylsiloxane (PDMS) films. Consequently, both the open-circuit voltage and short-circuit current density were significantly improved; notably, for the composite PDMS film containing 10 vol% SrTiO_3_ nanoparticles (NPs), the peak voltage and current density reached values of 305 V and 7.18 µA·cm^−2^, respectively. In 2017, Seung Wanchul et al. [43] fabricated a TENG featuring electrically controllable polarization along with robust triboelectric charge transfer characteristics by integrating a nanocomposite comprising high-dielectric ceramic BaTiO_3_—known for its excellent charge trapping ability—and a ferroelectric copolymer matrix poly(vinylidene fluoride-co-trifluoroethylene) (PVDF-TrFE). The following year, in 2018, Fang Zhenggang et al. [44] embedded particles of high-dielectric-constant CaCu_3_Ti_4_O_12_ (CCTO) into the PDMS matrix to further augment its dielectric properties. For comparative analysis, planar PDMS samples alongside PDMS with FTO patterned structures and those incorporating BaTiO_3_ within FTO patterns were utilized. Following the introduction of CCTO particles and surface textures into these composites, the output voltage from the TENG was approximately measured at 390 V (peak-to-peak), while the short-circuit current density reached around 170 mA m^−2^ with an associated charge density near 108 μC m^−2^. Additionally, in 2018, Park Hyun-Woo et al. [45] incorporated high dielectric constant TiO_2−x_ nanoparticles based on varying weight ratios to enhance output performance in PDMS-based TENGs. Notably, embedding just 5% NPs within PDMS yielded optimal results regarding both output voltage and current.

**Figure 4:**
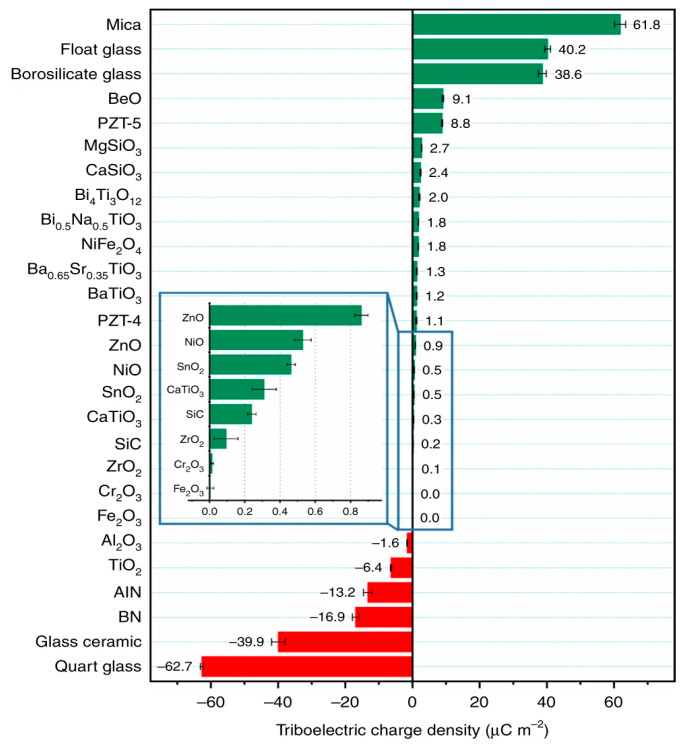
Chart of rankings of oxide materials and ferroelectric materials.

Based on the research published by Haiyang Zou [46] and others from Wang Zhonglin’s team [47] in *Nature Communications*, the triboelectric series, including metal oxide materials and ferroelectric materials, is ranked as shown in Figure 4.

In the application of carbon nanotube materials within the manufacturing domain of triboelectric nanogenerators (TENGs), carbon nanotubes are particularly well-suited for use as conductive layers due to their exceptional electrical and mechanical properties. Consequently, Zheng et al. [48] developed a stretchable multifunctional composite dielectric film by integrating a polyurethane (PU) matrix with carbon nanotubes (CNTs) and Fe_3_O_4_ nanoparticles. The CNTs embedded in the PU matrix create a network microstructure that enhances the contact area between the two triboelectric materials. Additionally, an increase in the content of high-dielectric-constant carbon nanotubes significantly expands the conductive pathways within the PU matrices. As a result, this leads to a substantial increase in the surface charge density of the CF-TENG composite material, thereby achieving improved output performance for TENGs.

Organic polymer materials are extensively employed across a myriad of domains, including petrochemicals, packaging, and military applications, owing to their advantageous properties, such as low density, excellent electrical insulation, corrosion-resistance, elasticity, and ease of processing. Presently, polymers have emerged as prevalent dielectric layer materials in triboelectric nanogenerators (TENGs). In 2021, Rana et al. [49] introduced poly-DADMAC into the positively charged triboelectric layer of nylon-11. The dipoles generated through this doping interacted synergistically with the functional groups present in nylon-11, thereby significantly enhancing the performance metrics of the TENG. This doped poly-DADMAC was effectively segregated from nylon-11—each exhibiting distinct dielectric characteristics—which culminated in the formation of a nanocapacitor model that augmented the dielectric constant of the composite dielectric layer. Similarly, in 2021, Wang et al. [50] incorporated polytetrafluoroethylene (PTFE) nanoparticles into the dielectric matrix of thermoplastic polyurethane (TPU), engendering a rich interfacial interaction between these two materials.

Two-dimensional (2D) materials, particularly M-Xene, represent a novel class of metal carbide/nitride compounds that exhibit remarkable mechanical properties, an extensive specific surface area, diverse surface functional groups, and exceptional electrical conductivity. These attributes render M-Xene highly advantageous for enhancing the surface charge density of dielectric layers, thereby significantly improving the performance of triboelectric nanogenerators (TENGs [51,52,53]). In 2021, Rana et al. [54] successfully developed a TENG (EN-TENG) by incorporating M-Xene into polyvinylidene fluoride–trifluoroethylene (PVDF-TrFE) composite films through an electrospinning technique. Their investigation encompassed both theoretical and experimental analyses to elucidate the impact of dielectric properties on the output performance of EN-TENG. Notably, when subjected to an optimal external load resistance of 4 MΩ, the maximum power density achieved by the fabricated EN-TENG reached an impressive 4.02 W/m^2^. The integration of PVDF-TrFE/M-Xene nanocomposites resulted in a fourfold enhancement in the output performance of EN-TENGs.

### 2.2. Surface Morphology Structuring and Chemical Functionalizing

**Figure 5:**
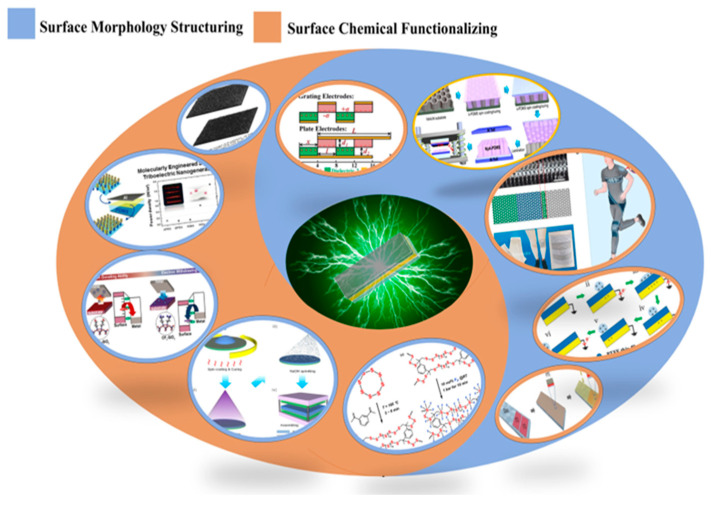
Diagrams of case of surface morphology construction and chemical functionalization.

Many researchers have concentrated on enhancing the power generation output performance of TENGs, particularly in L-S TENGs, through surface modification techniques [55]. Generally, their investigations have focused on two primary aspects: the preparation of surface microstructures and the chemical functionalization of surfaces, with fluorination being the most prevalent method. These efforts underscore the intrinsic relationship between interfacial dynamics and energy conversion processes, which is essential for elucidating interface reaction mechanisms, such as charge transfer and changes in electronic structure. This understanding is vital for optimizing the performance of L-S TENGs and guiding the design of more efficient triboelectric devices (shown in Figure 5).

The surface morphology and structure of triboelectric nanogenerators (TENGs) are critical determinants of their output performance. Surface modification can significantly enhance the effective contact area, thereby improving energy output. This enhancement is attributed to the dynamic behavior of droplets; hydrophobic surfaces, particularly superhydrophobic ones, exhibit advantages due to their water repellency and reduced lateral friction. These characteristics contribute to improved charge transfer efficiency and diminished charge dissipation. The dynamic behavior of droplets on the dielectric layer—including spreading, bouncing, and sliding—is closely linked to charge generation in L-S TENGs. Furthermore, the contact time and maximum spreading diameter of droplets are pivotal factors influencing this process Surface roughness and microstructure play a crucial role in determining both the generation and distribution of triboelectric charges. Hierarchical structures (Cassie–Baxter-type) have been shown to be more effective than Wenzel-type surfaces in enhancing durability and stability. However, an intriguing phenomenon arises when excessive roughness leads to a reduction in contact area and overall output [56].

In their 2014 study, Niu et al. [57] presented the initial theoretical model of grating structure TENGs and thoroughly examined two types of grating structure TENGs: equal-plate-length grating structure TENGs and unequal-plate-length grating structure TENGs. This analysis aimed to elucidate the disparities in their output characteristics. It was noted that grating and layered structures can improve the electrical output of TENGs by enabling multiple in-plane charge separation cycles and increasing the specific surface area. These structures are particularly effective in applications requiring high sensitivity and precision, such as self-powered sensors. Dudem, Bhaskar et al., 2017 [58] optimized the structure size of nanopillar structures distributed on the surface of PDMS (NpAs) structural dimensions to improve the output performance of TENGs. The present study investigates the effect of altering the period and diameter of NpAs on the PDMS surface on the output performance of TENG, both theoretically and experimentally. The optimal NpA-PDMS period and diameter of 125 nm and 60 nm were obtained. The TENG, when equipped with the aforementioned NpA-PDMS, exhibited an open-circuit voltage (VOC) and a short-circuit current of 568 V and 25.6 μA, respectively, at push frequencies of 10 N and 5 Hz. Yang, Lei, et al., in 2019, a robust working mechanism was proposed, taking into account the spreading, bouncing, sliding, and agglomeration of the droplets on solid surfaces during friction electrical cycling [59]. This mechanism was measured using the electrical output of a droplet-driven TENG (Wd-TENG) and observed with the help of a high-speed camera. The outcomes demonstrate that decreasing the contact duration of the droplets on a superhydrophobic surface enhanced the output performance of the L-S TENG. The macroscopic texture of the surface could further reduce the contact time and improve the energy harvesting efficiency. In a similar vein, Xu et al. [60] reported a novel TENG design inspired by the strong hydrophobicity and low sliding friction of porcupine. This design, termed SLIPS-TENG, utilizes lubricant-impregnated porous surfaces (SLIPS), and it exhibits numerous advantages over conventional designs in a variety of operating environments, including optical transparency, configurability, self-cleaning, flexibility, and power generation stability. In 2020, Dong et al. [61] reported a t-TENG with a spherical structure capable of efficiently harvesting energy from various mechanical sources, which can be seamlessly knitted into garments. The t-TENG is composed of two knitted layers that are alternately parallel and cross each other at the joints, and it is knitted directly on a computerized flat knitting machine. In 2024, Wu, Lingang, et al. [62] systematically summarized the morphology engineering and structural design strategies of t-TENGs by analyzing the working principle of t-TENGs. The presentation also included emerging applications of TENGs with specific structures and surfaces, as well as potential future development and industrial applications of TENGs.

The surface chemical functionalization of dielectric materials, particularly through fluorination, has been demonstrated to enhance the energy output of triboelectric nanogenerators (TENGs). This enhancement has been observed to result in increased hydrophobicity and friction electronegativity in polymeric materials. Researchers have adsorbed or tuned chemical functional groups to enhance the surface friction charge of frictionally charged materials through various process methods, such as the self-assembled monomolecular layer (SAM) method and ultraviolet ozone (UVO) irradiation. The advantage of surface chemical functionalization is that it can be used to increase the surface charge density by introducing chemical groups that have a tendency to gain or lose electrons. This strategy has been shown to significantly enhance the performance of TENG devices. The combination of chemical functionalization with material optimization and morphological aspects offers several advantages, particularly in the context of dielectric materials. The utilization of chemical functionalization enables the employment of a wider range of polymers in TENGs, facilitated by chemical modifications that can complement other approaches. This approach also provides hydrophobicity and allows for greater flexibility in the structural design of TENGs. Moreover, the selection of materials with stronger chemical bonds for commercial use can improve their durability. Halogens are the most electronegative preferred groups with high electron affinity; they are particularly well suited to accommodate additional electrons.

As early as 1996, Ulman, Abraham, et al. [63] advanced a two-pronged rationale for the efficacy of these SAMs. Firstly, they identified the elimination of moisture-sensitive alkyltrichlorosilanes from self-assembled monolayers (SAMs) as a pivotal factor. Secondly, they emphasized the strategic utilization of crystalline gold surfaces. This discourse was further enriched by the extensive experience of the Kuhn Laboratory in Göttingen, which had been engaged in the application of chlorosilane derivatives for hydrophobicity in glasses for many years. Additionally, it highlighted the meticulous preparation of SAMs of alkyl mercaptan salts on gold by the adsorption of di-n-alkyl disulfide in dilute solution, a technique pioneered by Nuzzo and Allara, et al. two significant factors contributing to their success. As stated by Hozumi, Atsushi, et al., 1999 [64], water-resistant surfaces can be prepared by exposing silicon substrates with surface oxides as hydroxyls to fluoroalkylsilane (FAS) vapors. In 2015, Song, Giyoung, et al. [65] introduced a self-assembled monomolecular layer (METS)-based molecularly engineered surface friction electric nanogenerator. The device varies the terminal functional groups of the self-assembled monomolecular layer (SAM) (using fluorine termination), which allows for facile control of the frictionally charged surface charge density of the substrate. When subjected to relatively mild mechanical contact with a vertical force of 3 N at 1.25 Hz, the device exhibited an open-circuit voltage of 105 V and a short-circuit current of 27 μA. The power density of the device was determined to be 1.8 W/m2 at a load resistance of 10 MΩ, which is more than 60 times higher than that of the unmodified dielectric/Al device. In the same year, Yun, Byung Kil, et al. [66] reported that the triboelectric surface charge of polydimethylsiloxane (PDMS) could be significantly enhanced simply by applying a sodium hydroxide (NaOH) solution. After spraying a 1 M NaOH solution onto the PDMS surface, the resulting triboelectric nanogenerator (TENG) produced a voltage of 10.4 V and a current of 179 nA. When PDMS was subjected to ultraviolet ozone treatment prior to the application of the NaOH solution, the generated triboelectric voltage and current increased to 49.3 V and 1.16 μA, respectively—values that were fifteen times greater than those observed for fresh PDMS. The conclusion drawn from these findings is that the increase in polar Si-O bonds, rather than a decrease in non-polar Si-CH_3_ bonds within PDMS, accounts for this enhancement in performance. In 2016, Byun, Kyung-Eun, et al. [67] systematically regulated the triboelectric polarity of the SiO_2_ layer surface by incorporating various electron-donating functional groups (including -NH_2_, -SH, and neutral group -CH_3_) and an electron-accepting functional group (-CF_3_). They demonstrated that these functional groups significantly influenced the surface dipole and electronic state, thereby controlling both the quantity and polarity of the triboelectric charge. Chunhua, et al. [68], 2017, developed a high-performance triboelectric nanogenerator (TENG) using contact materials composed entirely of cellulose nanofibrils (CNFs). They chemically modified cellulose molecules by attaching nitro and methyl groups to alter the triboelectric polarity of CNF, resulting in a substantial enhancement in its triboelectric output. The findings revealed that nitro CNFs exhibited a negative surface charge density of 85.8 µC m^−2^ while methyl CNFs displayed a positive surface charge density of 62.5 µC m^−2^; these values corresponded to approximately 71% and 52% of that observed for perfluoroethylene (FEP), respectively. In 2019, Lee, Jong Hyeok, et al. [69] reported the first instance of triboelectric energy harvesting utilizing a sulfur-based inorganic polymer synthesized through an inverse vulcanization process involving elemental sulfur—a by-product of petroleum refining. Compared with commercial PTFE films, fluorinated polymeric sulfur demonstrated an increase in triboelectric energy output (voltage and current) of sixfold and threefold respectively. The open-circuit voltage output reached as high as 1366 V and successfully powered 630 LEDs with a minimum applied force of approximately 30 N. In 2023, Shanbedi, Mina et al. [70] highlighted, in their comprehensive review article on improving the performance of TENGs, that the introduction of highly electronegative chemical groups, such as halogens, effectively enhances the triboelectric negativity of polymers, as supported by a summary of relevant case studies.

## 3. Experimental Research Combining Doped Materials and Surface Modification

**Figure 6:**
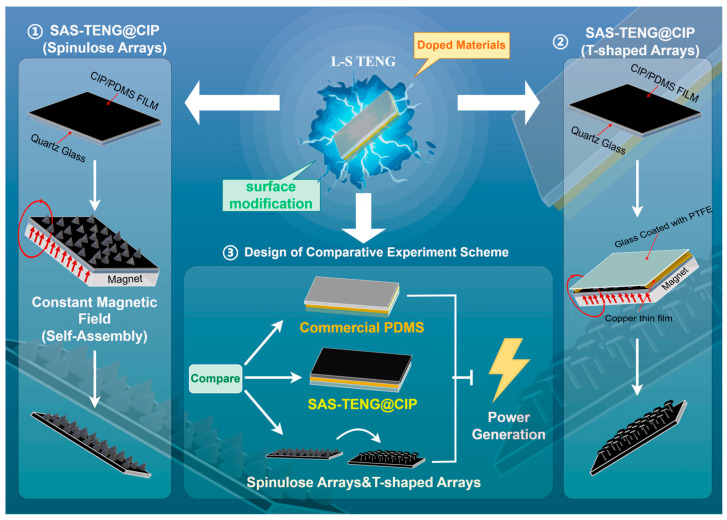
Schematics of designs of this experimental research.

As discussed in the preceding sections, numerous studies have been carried out to enhance the energy generation of TENGs. It is evident that prior research has predominantly focused on dielectric material doping and surface modification individually. However, investigations into the synergistic application of these two methods for improving TENG energy output remain scarce. Notably, a wealth of existing experimental data and theoretical analyses confirm that both dielectric material doping and advanced surface modification techniques can effectively enhance the energy output of various TENGs, including L-S TENGs. Nevertheless, it remains unclear which of these two processes exerts a greater influence on the power generation mechanism of TENGs, particularly L-S TENGs. Are these two manufacturing processes entirely independent, or do they interact with each other? If so, which process plays a more dominant role in enhancing TENG power generation? These questions warrant further exploration and clarification. Inspired by the dual approaches of dielectric material doping (mixing metals and metal oxides) and superhydrophobic surface microstructure self-assembly, this study proposes two types of Self-Assembled Surface TENG@Carbonyl Iron Particles Doping (SAS-TENG@CIP). These structures, featuring superhydrophobic surfaces, are simply fabricated by mixing CIP (primarily composed of iron and iron oxide) with dimethylsiloxane (PDMS) under a constant magnetic field, as illustrated in Figure 6.

Through the integration of interesting experimental findings with profound theoretical insights, this paper delves into the pivotal role played by the doping and surface modification processes of dielectric materials in amplifying the power generation capabilities of L-S TENGs. Furthermore, this study elucidates that both material doping and hydrophobic surface modification exhibit an inherent threshold. Beyond this threshold, their ability to enhance the power output plateau of L-S TENGs not only ceases to increase, but may also lead to a notable decline. To fully exploit the synergistic potential of these two approaches, a more profound comprehension of the underlying scientific mechanisms is essential. Such understanding facilitates their sophisticated integration and enhances power generation efficiency to an unprecedented extent.

### 3.1. Materials and Experimental Methods

As illustrated in Figure 6, the microstructure of the dielectric material layer was fabricated by referencing the work of Ge Chen et al. [71], which enabled the formation of spinulose arrays on the surface. In contrast, the fabrication of T-shaped surface arrays was inspired by the innovative design concepts proposed by Hujun Wang et al. [72].

This research experiment employed a variety of materials, including carbonyl iron nanoparticles (CIP) with diameters ranging from 5 to 50 nanometers (purchased from Sigma-Aldrich, St. Louis, MO, USA), SYLGARD 184 polydimethylsiloxane (PDMS) and its crosslinking curing agent (also purchased from Sigma-Aldrich, St. Louis, MO, USA), an oil-based PTFE coating solution (obtained from Alibaba, Hangzhou, China), a sintered neodymium–iron–boron block permanent magnet with a remanence of 1.42 Tesla and dimensions of 5 cm × 5 cm × 2.4 cm (from Alibaba, Hangzhou, China), a pure copper film 0.2 cm thick (Alibaba, Hangzhou, China), deionized water (acquired from Watsons, HongKong, China), copper wire with a diameter of 2 mm, and glass sheets (both obtained from Alibaba, Hangzhou, China).

**Figure 7:**
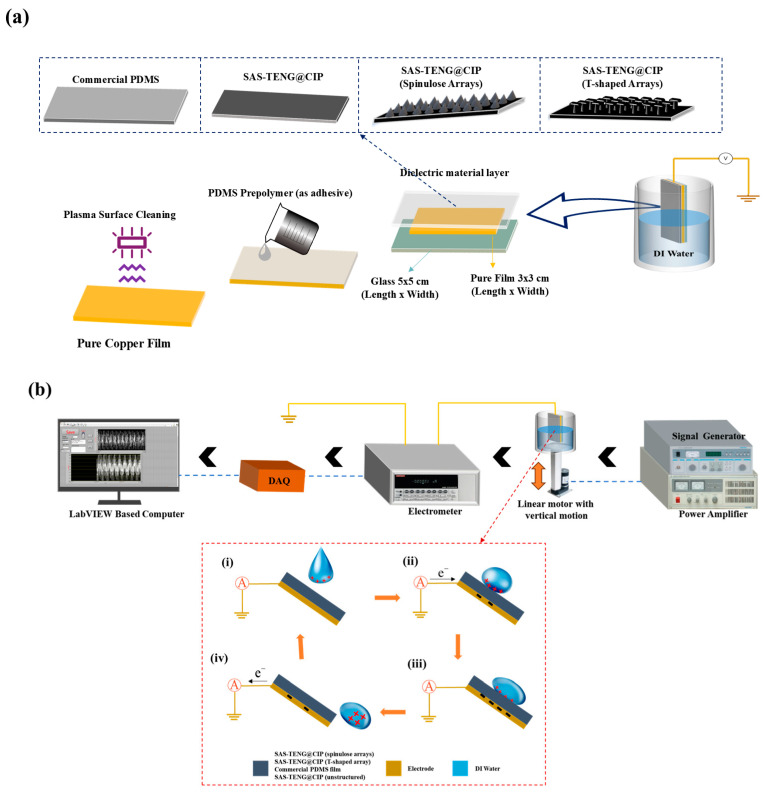
The materials, manufacturing processes, and experimental design of SAS-TENG@CIP.

As shown in Figure 7, the manufacturing process of the SAS-TENG@CIP involves several key steps. First, a 5 cm × 5 cm quartz glass sheet was cut to serve as the base substrate. To ensure the device’s sealing performance when in contact with deionized water, a 3 cm × 3 cm pure copper film was also cut and attached to the substrate. This design prevents the contamination of the pure copper conductive electrode by the liquid working medium during operation. The selection of the pure copper film aligns with the preferences outlined in Table 1, where most TENG devices utilize Cu electrodes among single-element metals due to their ability to facilitate efficient electron transfer from dielectric materials. Subsequently, the pure copper film was rigorously cleaned using a plasma surface cleaning device (FemtoScience, Hwaseong-si, Republic of Korea; 70 W power, 50 Hz frequency, 5 min cleaning time) to maintain its high conductivity. Before assembling the various surface microstructure-modified dielectric material layers onto the topmost layer of the SAS-TENG@CIP, PDMS and the curing agent were mixed according to the ratios specified in references. The mixture was evenly coated onto the glass sheet adhered with the pure copper film to minimize the distance between the dielectric layer and the conductive metal layer. The assembly was then firmly pressed using weights to ensure proper adhesion. Finally, all components were assembled and placed on a hot plate for 5 h to achieve complete sealing of the SAS-TENG@CIP devices.

In the selection of the L-S TENG configuration, the experimental design was based on the traditional single-electrode configuration. In this setup, charges are transferred from the positive friction layer to the negative friction layer, and the output voltage is measured between the positive friction layer (serving as the electrode) and a grounded electrode. To evaluate the experimental design for combining doped materials and surface modification, this study employed multiple pieces of equipment. Specifically, a signal generator and a power amplifier provided two synchronized signals to drive the linear motor at a fixed frequency of approximately 2 Hz for vertical motion. During the experiment, deionized water was placed in an open container, which was positioned on the platform at the end of the linear motor. Upon applying current and activating the response switch, the linear motor caused the rod to move vertically up and down, enabling continuous contact between the four types of TENG devices described in this paper and the liquid dielectric material. The generated charges were then conducted through the copper film and wires to the electrometer for measurement. Finally, the electrical signals were recorded using a data acquisition device installed on a computer equipped with LabView software (version 2024).

### 3.2. Interesting Results of Experiments Combining Doped Materials with Surface Modification

This study investigates which of the two processes—doping and surface modification of dielectric materials discussed earlier—has a greater influence on the power generation mechanism of TENGs, particularly L-S TENGs. It also examines whether these processes are entirely independent or exhibit synergistic interactions, as well as which process contributes more significantly to enhancing TENG power generation.

**Figure 8:**
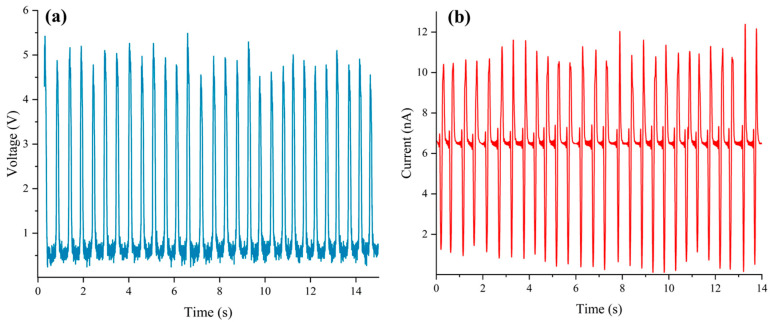
The power generation performance of commercial PDMS film. (**a**) Commercial PDMS in deionized water generates approximately 5.5 V. (**b**) commercial PDMS in deionized water generates approximately 10 nA.

**Figure 9:**
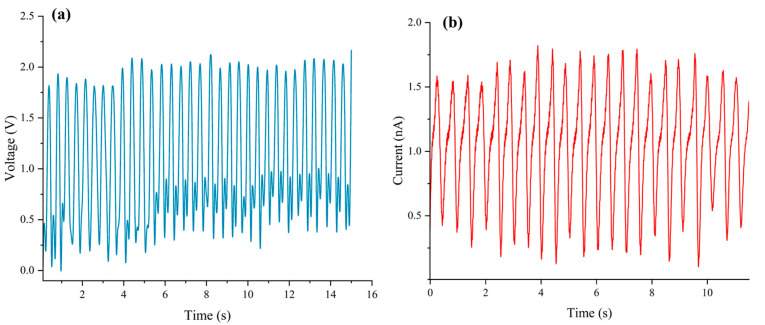
The power generation performance of SAS-TENG@CIP (unstructured). (**a**) SAS-TENG@CIP under the triboelectric effect produces approximately 1.6 V. (**b**) SAS-TENG@CIP under the triboelectric effect produces approximately 1.2 nA.

**Figure 10:**
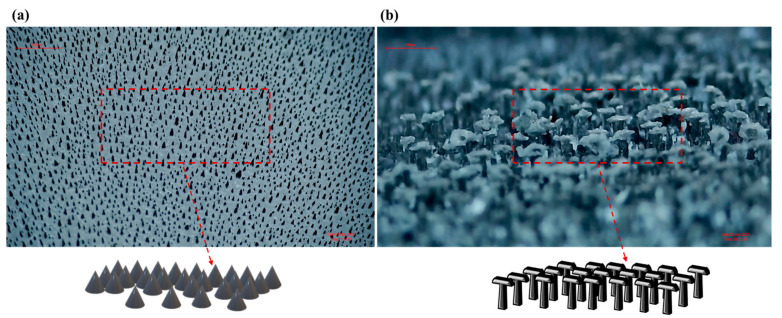
(**a**) Images of spinulose arrays. (**b**) T-shaped arrays.

**Figure 11:**
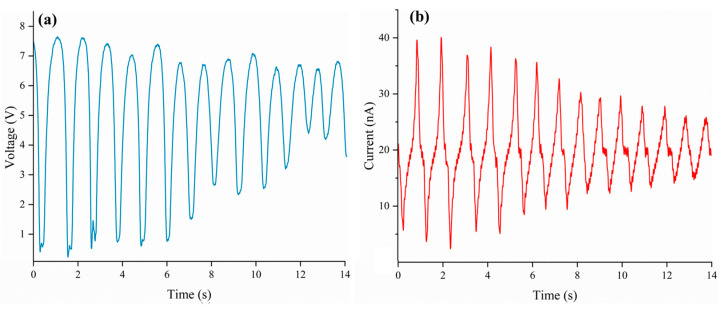
The power generation performance of SAS-TENG@CIP (spinulose arrays). (**a**) Voltage. (**b**) Current.

**Figure 12:**
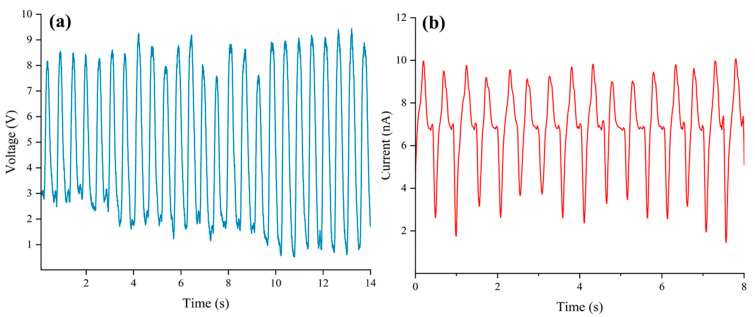
The power generation performance of SAS-TENG@CIP (T-shaped array). (**a**) Voltage. (**b**) Current.

Figure 8, Figure 9, Figure 10, Figure 11 and Figure 12 illustrate a series of notable power generation results from the experimental design. All experiments were performed at a frequency of 2 ± 0.5 Hz. As shown in Figure 8a,b, commercial PDMS in deionized water generates approximately 5.5 V and 10 nA via the triboelectric effect. As depicted in Figure 9a,b, SAS-TENG@CIP under the triboelectric effect produces approximately 1.6 V and 1.2 nA in deionized water.

It is worth noting that the TENG doped with CIP particles (primarily composed of iron and iron oxide) is theoretically expected to exhibit higher output voltage and current compared with commercial PDMS. However, the experimental data indicate the opposite trend.

Furthermore, can the surface modification process function independently of the dielectric material doping process and effectively address the decline in power generation performance observed in Figure 9a,b. To investigate this, the present study employed two types of SAS-TENG@CIP structures: one with a spinulose array micro-morphology (SEM image of the surface microstructure shown in Figure 10a) and the other with a T-shaped array micro-morphology (SEM image of the surface microstructure shown in Figure 10b) for power generation performance evaluation.

As illustrated in Figure 11a,b, when SAS-TENG@CIP (spinulose arrays) was employed, its power generation performance rapidly improved and temporarily surpassed that of commercial PDMS microdielectric materials. The output voltage ranged from 1 to 7 V, and the output current ranged from 10 to 40 nA. Nevertheless, this performance soon decreased to a level comparable to that of TENG doped with CIP particles, albeit slightly higher.

As depicted in Figure 12a,b, when SAS-TENG@CIP with T-shaped arrays is employed, its power generation performance exhibits a certain degree of recovery. The output voltage ranges from x to x volts, and the output current ranges from x to x nanamperes. While a gradual decay is still observed, the rate of decline is relatively moderate.

## 4. Discussion of Results and Theoretical Analysis

After the performance tests of four solid–liquid triboelectric nanogenerators (L-S TENGs) with SAS-TENG@CIP, SAS-TENG@CIP (spinulose arrays), SAS-TENG@CIP (T-shaped arrays), and commercial PDMS as dielectric materials were conducted based on the design model of a single-electrode L-S TENG and the combined method of material doping and surface modification, a series of interesting experimental results as shown in Figure 8, Figure 9, Figure 10, Figure 11 and Figure 12 were obtained. Referring to the theoretical research on composite dielectric materials by Dang, Zhi-Min, et al. [73], Nan Zhang [74], Kikuo Wakino [75], Yu BaoSheng, and Xiangyuan Wang, et al. [76], and strongly inspired by the research on the surface electrostatic breakdown (EB) effect of L-S TENGs published by Zhonglin Wang’s team in February of this year [77], theoretical analyses were respectively conducted on Figure 8, Figure 9, Figure 11 and Figure 12. Finally, some conclusions were drawn based on all the experimental data.

**Figure 13:**
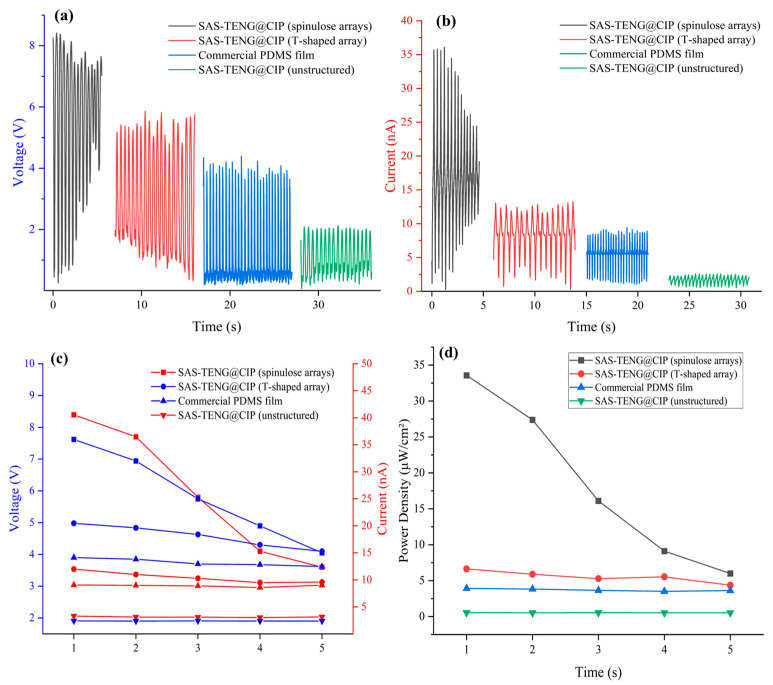
A comparative analysis of the power generation performance of four dielectric materials. (**a**) Voc (open-circuit voltage) comparison for four dielectrics; (**b**) Isc (short-circuit current) comparison for four dielectrics; (**c**) Line graph comparing the voltage and current data; (**d**) Line graph illustrating the comparison of the power density data.

As shown in Figure 13a–d, it is evident that the four dielectric materials: SAS-TENG@CIP, SAS-TENG@CIP (spinulose arrays), SAS-TENG@CIP (T-shaped ar-rays), and commercial PDMS—exhibit distinct power generation capabilities under identical experimental conditions. Specifically, Figure 13a,b reveal that, for both open-circuit voltage (VOC) and short-circuit current (ISC), the SAS-TENG@CIP (spinulose arrays) solid–liquid triboelectric nanogenerator demonstrates the highest power generation performance, but also experiences the fastest decline in output. The SAS-TENG@CIP (T-shaped arrays) ranks second in terms of power generation, with a mild decrease in output over time. The commercial PDMS-based solid–liquid triboelectric nanogenerator maintains stable power generation throughout. In contrast, the SAS-TENG@CIP type exhibits very weak power generation performance.

From the analysis of experimental data presented in Figure 13c,d, it is evident that the three types of experimental data: open-circuit output voltage (VOC), short-circuit output current (ISC), and unit energy density output are ranked in the following order: SAS-TENG@CIP (spinulose arrays)-type solid–liquid triboelectric nanogenerator, SAS-TENG@CIP (T-shaped arrays)-type solid–liquid triboelectric nanogenerator, commercial PDMS-type solid–liquid triboelectric nanogenerator, and finally, the SAS-TENG@CIP type-solid–liquid triboelectric nanogenerator.

In this chapter, the electrical percolation theory is employed to elucidate the weak power generation capability of the SAS-TENG@CIP-type solid–liquid triboelectric nanogenerator. Specifically, it is demonstrated that the excessive doping of CIP particles in this nano-generator results in a complex dielectric constant (DK), thereby causing charge leakage.

This chapter also employs the electrical percolation (EP) theory and the electrostatic breakdown (EB) theory (Wang Zhonglin team, 2025 [77]) to elucidate the phenomenon of enhanced and subsequently attenuated power generation capacity in the SAS-TENG@CIP (spinulose arrays) and SAS-TENG@CIP (T-shaped arrays) solid–liquid triboelectric nano-generators. Specifically, it explains the combined effects of excessive CIP particle doping, which leads to a complex dielectric constant (DK) and charge leakage, as well as electro-static breakdown (EB) occurring on the superhydrophobic surface.

### 4.1. Theory of Electrical Percolation in Doped Dielectric Materials

By comparing the results presented in Figure 8 and Figure 9, it is evident that the power generation capability of SAS-TENG@CIP has decreased substantially. According to the literature, the derivation formula for the charge density σ of the dielectric layer in triboelectric nanogenerators is provided. As illustrated in Figure 13, for an L-S TENG with no doped dielectric material (using PDMS as the dielectric layer), the formula for its capacitance C_1_ is given in Equation (1).

(1) $$C1=\frac{\varepsilon_{PDMS}\cdot s}{d}$$

where $\varepsilon_{PDMS}$ represents the dielectric constant of PDMS, s represents the electrode area, and d represents the thickness of the PDMS layer.

**Figure 14:**
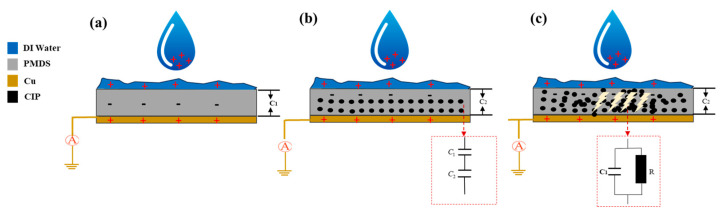
The mechanism of equivalent capacitance and electrical percolation. (**a**,**b**) The equivalent capacitance of a composite material (PDMS@CIP); (**c**) Diagram of the mechanism of electrical percolation.

When carbonyl iron nanoparticles (CIPs) with varying diameters are doped into PDMS, the CIP layers can be equivalently treated as multiple conductive layers as illustrated in Figure 14b. Therefore, inserting CIPs arranged in a regular pattern between the capacitors can be analogized to connecting a virtual capacitor in parallel with the original capacitor, where the capacitance value of this virtual capacitor is given by Equation (2). Here, R represents the thickness of the CIP particle layer arranged in a regular pattern

(2) $$C_{\varepsilon }=\frac{\varepsilon \cdot s}{d-R}$$

Therefore, the composite material capacitor $C_{2}$ in Figure 14b can be expressed as Equation (3).

(3) $$C_{2}=\frac{\varepsilon_{com}\cdot s}{d-\partial }$$

where $\varepsilon_{com}$ represents the composite dielectric constant of the doped material, ∂ represents that the effective thickness of $C_{2}$ capacitor has decreased, because the parallel connection of the virtual capacitor $C_{\varepsilon }$ can be regarded as a reduction in the effective thickness of the original capacitor $C_{1}$.

According to Kikuo Wakino’s 1993 research [75], which simulated the dielectric constant of composed of two different materials using Monte Carlo and finite element methods, in the high concentration range of high-dielectric-constant materials (such as PDMS), the simulated values of the dielectric constant exhibit a trend toward the “parallel model”. By introducing the constant α, the type of doping mixing rule is determined. For instance, under the “parallel model”, the value of α is set to 1. This study asserts that the dielectric constant calculated using Equation (4) shows good agreement with the measured values.

(4) $$\varepsilon_{r}=\exp\left[\frac{\ln\left\{V_{1}\varepsilon_{r1}^{\left(V_{1}-V_{0}\right)}+V_{2}\varepsilon_{r2}^{\left(V_{1}-V_{0}\right)}\right\}}{V_{1}-V_{0}}\right]$$

where $\varepsilon_{r}$, $\varepsilon_{r1}$, and $\varepsilon_{r2}$ are the relative dielectric constants of the compound, material 1, and material 2. Respectively, $V_{1}$ and $V_{2}$ are the volume fractions of material 1 and material 2.

In the equation, $V_{0}$ denotes the critical volume fraction at the intersection point between the predicted dielectric constant curve and the logarithmic mixing rule. However, within the critical volume mixing region, the electric flux pattern exhibits considerable complexity. In most cases, the cluster-like configuration (a local complex combination of parallel and series model patterns) prevails. When $\alpha =V_{1}-V_{0}$, the calculated value of Equation (4) achieves the best fit with the results obtained using the Monte Carlo method. Based on the experimental findings regarding doping in cluster-like materials presented in this study, the dielectric constant of the dielectric material decreases when α is set to either −1 or 1.

In fact, there is a distinct relationship between the volume fraction of conductive particles and the conductivity of the composite formed by conductive particles and dielectric materials (e.g., PDMS). In this context, the “percolation phenomenon” plays a critical role in determining the electrical conduction behavior. Similar to the simulation results of the apparent dielectric constant presented in this study, as early as 1989, Yu BaoSheng, Xiangyuan Wang, et al. [76] demonstrated, through experiments on the dielectric constant regulation of CIP, that doping material particles into PDMS can alter the dielectric constant of the functional friction layer. According to the referenced report [76], the dielectric constant of the composite material layer can be determined.

Dielectric constant $\varepsilon_{com}$ of composite layer as Equation (5).

(5) $$\begin{array}{l} \varepsilon_{com}={\varepsilon_{PDMS}}^{A1}\cdot {\varepsilon_{CIP}}^{A2}\\ A1={V_{PDMS}}^{0.77},A2={V_{CIP}}^{0.77} \end{array}$$

Reference [76] indicates that, when $V_{CIP}$ = 30%, the dielectric constant $\varepsilon_{com}$ of the composite material made of carbonyl iron powder (CIP) and polymer materials will decrease to 60% of its original value.

(6) $$\sigma =\frac{\left[\left(W-E^{\prime }\right)/e\right]\left(1+d/\varepsilon_{com}\cdot x\right)}{d/\varepsilon_{0}\varepsilon_{com}}$$

where W denotes the electrical power of the metal component, $\varepsilon_{0}$ presents the dielectric constant in a vacuum, $E^{\prime }$ is the work function of the composite material film, and x is the gap distance between DI water and the composite material. Therefore, according to Equation (6), the dielectric layer charge density σ of SAS-TENG@CIP is strongly influenced by the dielectric constant $\varepsilon_{com}$ of the composite material, showing a numerical decrease. This theoretically explains the significant decline in the power generation performance of the device, as shown in Figure 9.

Furthermore, based on the theory referenced, the current transmission characteristics of the composite materials used in this experiment are significantly influenced by percolation theory. In this study, to ensure the self-assembly stability of spinulose arrays and T-shaped arrays, a relatively large proportion of CIP was doped, which would lead to a filler concentration higher than the percolation threshold. Consequently, the conductivity of the conductive filler–polymer composites would exhibit a nonlinear increase in dielectric value. This phenomenon cannot be explained by classical mixing rules, such as the Maxwell–Garnett equation, Bruggeman self-consistent effective medium approximation, Jaysundere–Smith equation, Lichtenker rule, and so on, but can only be explained by percolation theory. The intuitive explanation of percolation theory can be understood as follows: when conductive fillers are added to a polymer system, as the addition concentration increases, the conductive particles will aggregate with each other, resembling small islands. As the doping concentration continues to increase, the area of these conductive islands will also increase, gradually forming a conductive network. The critical value at this point is called the percolation threshold. The overall conductivity will be greatly enhanced compared with that before, and the final result is that the upper and lower electrodes will be conductively connected, forming a leakage current, increasing the dielectric loss, and the output power of the device in the external circuit will be greatly decreased.

Overall, this section investigates the dielectric properties of CIP-doped PDMS composite materials and their associated percolation of current phenomenon, as illustrated in Figure 14a–c. It elucidates the cluster structure phenomenon induced by the irregular arrangement of CIP particles, which brings the system close to the percolation threshold. This proximity results in a significant reduction in power generation capacity due to current percolation in the experiment. Equations (4)–(6) further elaborate on the influence of the percolation threshold theory on the dielectric constant and charge density of the dielectric layer through numerical simulations and experimental validation. In summary, it can be inferred that moderate doping of CIP in PDMS enhances its dielectric constant; however, excessive doping approaching the percolation threshold leads to leakage currents, thereby diminishing the device’s performance.

### 4.2. Theory of Electrostatic Breakdown (EB) on Surface Modification

**Figure 15:**
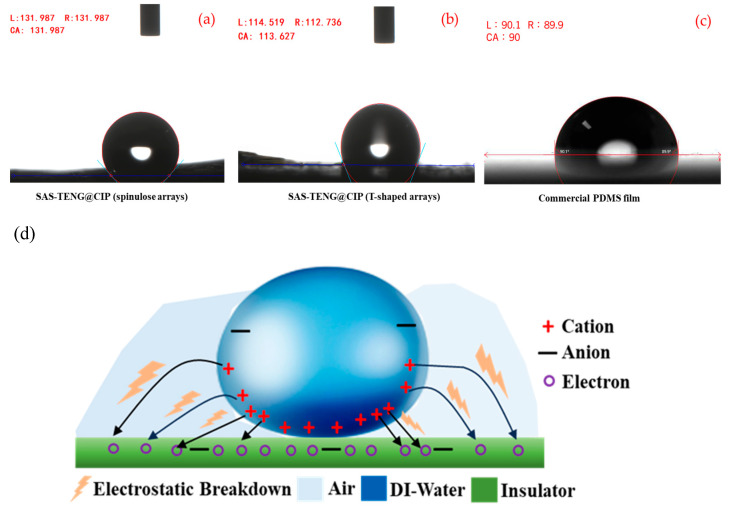
(**a**–**c**) The hydrophobicity characteristic maps of TENG@CIP (spike array) and SAS-TENG@CIP (T-shaped array) compared with commercial PDMS films, and (**d**) the diagram of Electrostatic breakdown (EB) mechanism [77].

In response to the power output attenuation phenomenon illustrated in Figure 9, this study developed two types of SAS-TENG@CIP devices with hydrophobic microstructured surfaces—SAS-TENG@CIP (spinulose arrays) and SAS-TENG@CIP (T-shaped arrays)—under a constant magnetic field force (as shown in Figure 15). These were designed to mitigate the performance degradation attributed to percolation theory. Figure 11 presents the power generation performance of SAS-TENG@CIP (spinulose arrays), while Figure 12 illustrates the performance of SAS-TENG@CIP (T-shaped arrays). It is evident that the hydrophobic surface enhances the capture of positive charges from deionized water by the dielectric layer, thereby exerting a noticeable short-term compensatory effect.

However, it is surprising to note that the compensation mechanism exhibits significant instability, particularly for SAS-TENG@CIP (spinulose arrays), which demonstrates pronounced signs of rapid attenuation. Although the power generation output of SAS-TENG@CIP (T-shaped arrays) improves in a relatively stable manner, the compensatory effect on power generation performance does not meet the expected level.

The study in Reference [77] on the electrostatic breakdown (EB) effect at the surface of L-S TENGs has provided critical insights (as shown in Figure 15d). According to its analysis, the power generation loss caused by electrostatic breakdown (EB) near the liquid–solid–gas interface can be attributed to the intensified electric field in the gas region close to the three-phase contact line. During the continuous impact of water droplets on the insulator surface, negative charges progressively accumulate on the insulator until reaching saturation. Once the electric field strength near the liquid–solid–gas interface surpasses the threshold for gas breakdown, a sudden discharge phenomenon occurs.

In addition, Wang’s team also highlighted, in their research on the EB effect, that:

(1) A conductive substrate on the solid surface can exacerbate local breakdown;
(2) Sharp tips or edges can further intensify breakdown and reduce the surface charge density.

The substantial amount of highly conductive material CIP and the sharp edges present in SAS-TENG@CIP (spinulose arrays) fully align with these two points, thereby providing an explanation for the peculiar phenomenon observed in Figure 11. In conjunction with percolation theory, this study hypothesizes that the strong coupling between the EB effect and the percolation effect may lead to the failure of SAS-TENG@CIP (spinulose arrays). For SAS-TENG@CIP (T-shaped arrays), it only partially satisfies one characteristic described in the aforementioned percolation theory and EB theory. Consequently, as shown in Figure 12, its power generation performance is unstable, and the improvement effect remains suboptimal.

## 5. Conclusions

Based on a systematic review and reclassification of the existing literature on enhancing the power generation performance of L-S TENG through composite materials and surface modification, this paper draws inspiration from the doping of dielectric materials (e.g., mixed metals and metal oxides), as well as the self-assembly approach for superhydrophobic surface microstructures. Consequently, an experimental design named “Self-Assembled Surface TENG@Carbonyl Iron Particles Doping (SAS-TENG@CIP)” is proposed, and a series of experiments are conducted to investigate the synergistic effects of doped composite materials and superhydrophobic surface modification on the power generation capacity of L-S TENG. Through a detailed analysis of the experimental data, the following conclusions are drawn in this paper:

(1) For all these “TENGs” considered as “special capacitors”, the selection of high-quality composite dielectric materials to minimize current leakage is more critical than solely concentrating on surface modification for power enhancement.
(2) For the enhancement of power generation performance in triboelectric nanogenerators (TENGs), various materials such as insulating polymers, ferroelectric substances, and conductive metals (including metal oxides) are employed as fillers for doping modifications. Each material possesses an inherent threshold; exceeding this threshold can result in a decline in the dielectric constant of high-filler-content composite materials due to weak interfacial interactions and an increase in internal porosity. According to percolation theory, excessive incorporation of conductive metal materials (including metal oxides) as fillers may compromise the internal structure of these “special capacitors” within TENGs. This disruption could lead to chaotic effects or structural failure, ultimately resulting in leakage phenomena (. Moreover, the ML-TENGPro power generation experiment in a fully emulsified liquid has provided some evidence to support the hypothesis that the (A/O/W) droplet structure model is a “charge storage-enhanced droplet”.
(3) For L-S TENGs, while hydrophobic surface modification is an effective approach to enhance power generation performance, it is important to note that, without adequate prevention of electrostatic breakdown (EB), this method alone will encounter an upper threshold limit. The magnitude of this threshold varies depending on the operational environment of L-S TENGs, such as air dryness and the charge carried by droplets during power generation. This variability significantly constrains the practical engineering applications of L-S TENGs. The term “prototype” has been used to refer to the ML-TENG in light of the experimental results presented in Conclusion. However, this does not negate the value of the theoretical insights provided in this paper, which can inform future developments in sensors for assessing the quality of lubricants in power systems.
(4) The performance degradation observed in SAS-TENG@CIP (flat), SAS-TENG@CIP (spinulose arrays), and SAS-TENG@CIP (T-shaped arrays) in this study can be attributed to the experimental validation of percolation theory and electrostatic breakdown (EB) theory. Notably, for SAS-TENG@CIP (spinulose arrays), the degradation is particularly significant due to the synergistic interaction of these two mechanisms.

With regard to future research, it is essential to fully harness the synergistic potential of doping and surface modification techniques in dielectric materials. This is particularly important for elucidating the underlying scientific mechanisms, especially regarding the determination of thresholds related to percolation theory and electrostatic breakdown (EB) phenomena. Such insights will facilitate their intricate integration and significantly enhance the power generation efficiency in forthcoming studies to an unprecedented level.

## Figures and Tables

**Table 1:** TENG device: operational mode, manufacturing materials, application fields, and output performance indicators.

Category of the Device	Mode	Dielectric Materials/Electrodes	Application	Output Performance
DB-TENG	S-L (Solid-liquid contact)	FEP/Cu	Energy Harvesting	V_OC_ = 44.0 V Isc = 52.02 nA
DEG	S-L	PTFE/ITO	Energy Harvesting	V_OC_ = 143.5 V Isc = 270 μA
WD-TENG	S-L	LDH/Al	Energy Harvesting	V_OC_ = 12.0 V Isc = 2.0 μA
LST-TENG	S-L	PTFE/Cu	Self-powered Sensor	V_OC_ = 40.0 V Isc = 15 nA
BSRW-TENG	S-L	BH-resin + BH-PDMS/Au	Self-powered Sensor	V_OC_ = 55.0 V Isc = 110 nA
Bacteria-Detection TENG	S-L	CNT-Arg/ITO-Van	Self-powered Sensor	V_OC_ = 155.0 V (*E. coli*) V_OC_ = 165.0 V (*P. aeruginosa*)
mSM-TENG	S-L	SMPU + PET/Al	Environmental Monitoring	V_OC_ = 150–320 V Isc = 2.5–4 μA
TENG(Gnb)	S-L	CNT-ConA/ITO-ConA	Environmental Monitoring	V_OC_ = 150.0 V (*S. aureus*) V_OC_ = 160.0 V (*P. aeruginosa*)
CG-TENG	S-S (Solid-Solid contact)	Silicone Rubber/C-coffee ground	Healthcare	V_OC_ = 150.0 V Isc = 2.1 μA
TENG (biocompatible bite sensor)	S-S	PVA/Cu	Healthcare	V_OC_ = 20.0 V Isc = 200 nA
TENGs Array	S-L	PTFE + PET/Cu	Corrosion Resistance	V_OC_ = 105.0 V Isc = 2.68 μA
S-TENG	S-S&S-L	Sponge + PTFE/Al + Cu	Corrosion Resistance	V_OC_ = 88.9 V Isc = 186 mA
FE-TENG	Contactless	PVDF + PDMS/ITO	Self-powered Sensor	0.85~1.23 mW
CDL-TENG	Contactless	Co-NPC(Ecoflex) + MXene(Ecoflex)/Conductive Fabric(Ag Covered)	Self-powered Sensor	V_OC_ = 21.0 V Isc = 9.85 μAm^−2^
TENG (high-performance)	Contactless	Siloxene(Ecoflex) + MoS_2_(LIG)/Cu	Self-powered Sensor	V_OC_ = 31.0 V Isc = 7.0 μAm^−2^
CTD	Contactless	Silicone Rubber + Nylon/Cu	Self-powered Sensor	V_OC_ = 0.212 Vm^−2^
NCTS	Contactless	PDMS/Al	Healthcare	V_OC_ = 200 mV
TENG (PVDF@Ti_3_C_2_T_x_ Spun Film-based)	Contactless	(PVDF)@Ti_3_C_2_T_x_ + PA/Cu	Self-powered Sensor	P_D_ = 200 μW/cm^2^ Q_nc_ = 128 μC/cm^2^

## Data Availability

Data are contained within the article.

## References

[1] Wang Z.L., Wang A.C. (2019). On the origin of contact-electrification. Mater. Today.

[2] Xu C., Zi Y., Wang A.C., Zou H., Dai Y., He X., Wang P., Wang Y., Feng P., Li D. (2018). On the electron-transfer mechanism in the contact-electrification effect. Adv. Mater..

[3] Lin S., Xu L., Xu C., Chen X., Wang A.C., Zhang B., Lin P., Yang Y., Zhao H., Wang Z.L. (2019). Electron transfer in nanoscale contact electrification: Effect of temperature in the metal–dielectric case. Adv. Mater..

[4] Cai C., Luo B., Liu Y., Fu Q., Liu T., Wang S., Nie S. (2022). Advanced triboelectric materials for liquid energy harvesting and emerging application. Mater. Today.

[5] Xiang T., Chen X., Sun H., Liu D., Jiang Y., Chen S., Xie Y., Zhang S. (2024). Advances in liquid-solid triboelectric nanogenerators and its applications. J. Mater. Sci. Technol..

[6] Nguyen Q.T., Vu D.L., Le C.D., Ahn K.K. (2023). Recent progress in self-powered sensors based on liquid–solid triboelectric nanogenerators. Sensors.

[7] Ahmed A.A., Qahtan T.F., Owolabi T.O., Agunloye A.O., Rashid M., Ali M.S.M. (2024). Waste to sustainable energy based on TENG technology: A comprehensive review. J. Clean. Prod..

[8] Wu Y., Zeng Q., Tang Q., Liu W., Liu G., Zhang Y., Wu J., Hu C., Wang X. (2020). A teeterboard-like hybrid nanogenerator for efficient harvesting of low-frequency ocean wave energy. Nano Energy.

[9] Wei X., Zhao Z., Zhang C., Yuan W., Wu Z., Wang J., Wang Z.L. (2021). All-weather droplet-based triboelectric nanogenerator for wave energy harvesting. ACS Nano.

[10] Liu G., Xiao L., Chen C., Liu W., Pu X., Wu Z., Hu C., Wang Z.L. (2020). Power cables for triboelectric nanogenerator networks for large-scale blue energy harvesting. Nano Energy.

[11] Xu W., Zheng H., Liu Y., Zhou X., Zhang C., Song Y., Deng X., Leung M., Yang Z., Xu R.X. (2020). A droplet-based electricity generator with high instantaneous power density. Nature.

[12] Cui P., Wang J., Xiong J., Li S., Zhang W., Liu X., Gu G., Guo J., Zhang B., Cheng G. (2020). Meter-scale fabrication of water-driven triboelectric nanogenerator based on in-situ grown layered double hydroxides through a bottom-up approach. Nano Energy.

[13] Xu M., Wang S., Zhang S.L., Ding W., Kien P.T., Wang C., Li Z., Pan X., Wang Z.L. (2019). A highly-sensitive wave sensor based on liquid-solid interfacing triboelectric nanogenerator for smart marine equipment. Nano Energy.

[14] Zhang X., Yu M., Ma Z., Ouyang H., Zou Y., Zhang S.L., Niu H., Pan X., Xu M., Li Z. (2019). Self-powered distributed water level sensors based on liquid–solid triboelectric nanogenerators for ship draft detecting. Adv. Funct. Mater..

[15] Li W., Lu L., Kottapalli A.G.P., Pei Y. (2022). Bioinspired sweat-resistant wearable triboelectric nanogenerator for movement monitoring during exercise. Nano Energy.

[16] Wang C., Wang P., Chen J., Zhu L., Zhang D., Wan Y., Ai S. (2022). Self-powered biosensing system driven by triboelectric nanogenerator for specific detection of Gram-positive bacteria. Nano Energy.

[17] Xiong J., Luo H., Gao D., Zhou X., Cui P., Thangavel G., Parida K., Lee P.S. (2019). Self-restoring, waterproof, tunable microstructural shape memory triboelectric nanogenerator for self-powered water temperature sensor. Nano Energy.

[18] Zhou Z., Wang P., Li J., Wang C., Chen J., Zhu L., Zhu H., Zhang D. (2022). A self-powered microbiosensor system for specific bacteria detection based on triboelectric nanogenerator. Nano Energy.

[19] Li M., Cheng W.-Y., Li Y.-C., Wu H.-M., Wu Y.-C., Lu H.-W., Cheng S.-L., Li L., Chang K.-C., Liu H.-J. (2021). Deformable, resilient, and mechanically-durable triboelectric nanogenerator based on recycled coffee waste for wearable power and self-powered smart sensors. Nano Energy.

[20] Panda S., Hajra S., Kim H.-G., Achary P.G.R., Pakawanit P., Yang Y., Mishra Y.K., Kim H.J. (2023). Sustainable solutions for oral health monitoring: Biowaste-derived triboelectric nanogenerator. ACS Appl. Mater. Interfaces.

[21] Sun W., Zheng Y., Li T., Feng M., Cui S., Liu Y., Chen S., Wang D. (2021). Liquid-solid triboelectric nanogenerators array and its applications for wave energy harvesting and self-powered cathodic protection. Energy.

[22] Wu M., Guo W., Dong S., Liu A., Cao Y., Xu Z., Lin C., Zhang J. (2022). A hybrid triboelectric nanogenerator for enhancing corrosion prevention of metal in marine environment. npj Mater. Degrad..

[23] Kim H.S., Kim D.Y., Kim J., Kim J.H., Kong D.S., Murillo G., Lee G., Park J.Y., Jung J.H. (2019). Ferroelectric-polymer-enabled contactless electric power generation in triboelectric nanogenerators. Adv. Funct. Mater..

[24] Rana S.M.S., Zahed M.A., Rahman M.T., Salauddin M., Lee S.H., Park C., Maharjan P., Bhatta T., Shrestha K., Park J.Y. (2021). Cobalt-Nanoporous Carbon Functionalized Nanocomposite-Based Triboelectric Nanogenerator for Contactless and Sustainable Self-Powered Sensor Systems. Adv. Funct. Mater..

[25] Shrestha K., Sharma S., Pradhan G.B., Bhatta T., Maharjan P., Rana S.S., Lee S., Seonu S., Shin Y., Park J.Y. (2022). A Siloxene/Ecoflex Nanocomposite-Based Triboelectric Nanogenerator with Enhanced Charge Retention by MoS2/LIG for Self-Powered Touchless Sensor Applications. Adv. Funct. Mater..

[26] Zhang W., Lu Y., Liu T., Zhao J., Liu Y., Fu Q., Mo J., Cai C., Nie S. (2022). Spheres Multiple Physical Network-Based Triboelectric Materials for Self-Powered Contactless Sensing. Small.

[27] Anaya D.V., Zhan K., Tao L., Lee C., Yuce M.R., Alan T. (2021). Contactless tracking of humans using non-contact triboelectric sensing technology: Enabling new assistive applications for the elderly and the visually impaired. Nano Energy.

[28] Xu Q., Gong J., Chen J., Zhang Y., Zhao H., Yin J., Zhao R., Lyu C., Ding W., Wu C. (2025). Contactless Triboelectric Sensing for Real-Time 3D Motion Recognition in Human-Computer Interaction. Adv. Electron. Mater..

[29] Wang Z.L. (2013). Triboelectric nanogenerators as new energy technology for self-powered systems and as active mechanical and chemical sensors. ACS Nano.

[30] Zi Y., Niu S., Wang J., Wen Z., Tang W., Wang Z.L. (2015). Standards and figure-of-merits for quantifying the performance of triboelectric nanogenerators. Nat. Commun..

[31] Zi Y., Wang Z.L., Yang Y., Zhai J., Wang J. (2023). Figure of Merit of Triboelectric Nanogenerator. Handbook of Triboelectric Nanogenerators.

[32] Zou H., Nguyen T.D., Pace G. (2025). Materials and figures of merit for nanogenerators. MRS Bull..

[33] Cho E., Jang H.S., Kim Y.Y., Yong H., Cho S.-P., Park J.-S., Myung J.S., Lee S.-J. (2024). Light and triboelectrification management by nanostructure coupled with plasma-polymerized-fluorocarbon thin film for enhancing performance of energy harvestings. Mater. Today Energy.

[34] Zhang Z., Chen Y., Debeli D.K., Guo J.S. (2018). Facile method and novel dielectric material using a nanoparticle-doped thermoplastic elastomer composite fabric for triboelectric nanogenerator applications. ACS Appl. Mater. Interfaces.

[35] Chen B.D., Tang W., Zhang C., Xu L., Zhu L.P., Yang L.J., He C., Chen J., Liu L., Zhou T. (2018). Au nanocomposite enhanced electret film for triboelectric nanogenerator. Nano Res..

[36] Biutty M.N., Koo J.M., Zakia M., Handayani P.L., Choi U.H., Yoo S.I. (2020). Dielectric control of porous polydimethylsiloxane elastomers with Au nanoparticles for enhancing the output performance of triboelectric nanogenerators. RSC Adv..

[37] Mu J.L., Han X.T., Yu J.B., Song J.S., He J., Geng W.P., Zou J., Xian S., Chou X.J. (2022). Magnetic levitation type double helix self-powered acceleration sensor based on ZnO-RTV Film. Adv. Mater. Technol..

[38] Bai Z., Xu Y., Li J., Zhu J., Gao C., Zhang Y., Wang J., Guo J. (2020). An eco-friendly porous nanocomposite fabric-based triboelectric nanogenerator for efficient energy harvesting and motion sensing. ACS Appl. Mater. Interfaces.

[39] Zhang H., Zhang P., Li P., Deng L., Zhang W., Liu B., Yang Z. (2022). Enhanced performance triboelectric nanogenerator based on porous structure C/MnO2 nanocomposite for energy harvesting. Nano Res..

[40] Gao L., Li J., Wang Z., Bu M., Zhai L., Wu S., Hu N., Dai K., Wu L., Lee A. (2022). A high performance triboelectric nanogenerator based on ordered doping technique for human-machine interaction sensing. Nano Energy.

[41] Chiang C.K., Popielarz R. (2002). Polymer composites with high dielectric constant. Ferroelectrics.

[42] Chen J., Guo H., He X., Liu G., Xi Y., Shi H., Hu C. (2016). Enhancing performance of triboelectric nanogenerator by filling high dielectric nanoparticles into sponge PDMS film. ACS Appl. Mater. Interfaces.

[43] Seung W., Yoon H., Kim T.Y., Ryu H., Kim J., Lee J., Lee J.H., Kim S., Park Y.K., Park Y.J. (2017). Boosting power-generating performance of triboelectric nanogenerators via artificial control of ferroelectric polarization and dielectric properties. Adv. Energy Mater..

[44] Fang Z., Chan K.H., Lu X., Tan C.F., Ho G.W. (2018). Surface texturing and dielectric property tuning toward boosting of triboelectric nanogenerator performance. J. Mater. Chem. A.

[45] Park H.-W., Huynh N.D., Kim W., Hwang H.J., Hong H., Choi K., Song A., Chung K.-B., Choi D. (2018). Effects of embedded TiO_2−x_ nanoparticles on triboelectric nanogeneratorperformance. Micromachines.

[46] Zou H., Guo L., Xue H., Zhang Y., Shen X., Liu X., Wang P., He X., Dai G., Jiang P. (2020). Quantifying and understanding the triboelectric series of inorganic non-metallic materials. Nat. Commun..

[47] Liu D., Yin X., Guo H., Zhou L., Li X., Zhang C., Wang J., Wang Z.L. (2019). A constant current triboelectric nanogenerator arising from electrostatic breakdown. Sci. Adv..

[48] Zheng J., Wei X., Li Y., Dong W., Li X., E S., Wu Z., Wen J. (2021). Stretchable polyurethane composite foam triboelectric nanogenerator with tunable microwave absorption properties at elevated temperature. Nano Energy.

[49] Rana S.M.S., Rahman M.T., Sharma S., Salauddin M., Yoon S.H., Park C., Maharjan P., Bhatta T., Park J.Y. (2021). Cation functionalized nylon composite nanofibrous mat as a highly positive friction layer for robust, high output triboelectric nanogenerators and self-powered sensors. Nano Energy.

[50] Wang H.L., Guo Z.H., Zhu G., Pu X., Wang Z.L. (2021). Boosting the power and lowering the impedance of triboelectric nanogenerators through manipulating the permittivity for wearable energy harvesting. ACS Nano.

[51] Khandelwal G., Maria Joseph Raj N.P., Kim S.J. (2021). Materials beyond conventional triboelectric series for fabrication and applications of triboelectric nanogenerators. Adv. Energy Mater..

[52] Gogotsi Y., Anasori B. (2019). The Rise of MXenes. ACS Nano.

[53] Bhatta T., Maharjan P., Cho H., Park C., Yoon S.H., Sharma S., Salauddin M., Rahman M.T., Rana S.M.S., Park J.Y. (2021). High-performance triboelectric nanogenerator based on MXene functionalized polyvinylidene ffuoride composite nanoffbers. Nano Energy.

[54] Rana S.M.S., Rahman M.T., Salauddin M., Sharma S., Maharjan P., Bhatta T., Cho H., Park C., Park J.Y. (2021). Electrospun PVDF-TrFE/MXene nanofiber mat-based triboelectric nanogenerator for smart home appliances. ACS Appl. Mater. Interfaces.

[55] Chen Z., Lu Y., Li R., Li D., Xiang B., Li J., Liu Q. (2023). Liquid-solid contact electrification through the lens of surface and interface science. Nano Energy.

[56] Kim K., Ahn S.K., Choi J.H., Yoo J., Eo Y.-J., Cho J.-S., Cho A., Gwak J., Song S., Cho D.-H. (2018). Highly efficient Ag-alloyed Cu (In, Ga) Se2 solar cells with wide bandgaps and their application to chalcopyrite-based tandem solar cells. Nano Energy.

[57] Niu S., Wang S., Liu Y., Zhou Y.S., Lin L., Hu Y., Pradel K.C., Wang Z.L. (2014). A theoretical study of grating structured triboelectric nanogenerators. Energy Environ. Sci..

[58] Dudem B., Huynh N.D., Kim W., Kim D.H., Hwang H.J., Choi D., Yu J.S. (2017). Nanopillar-array architectured PDMS-based triboelectric nanogenerator integrated with a windmill model for effective wind energy harvesting. Nano Energy.

[59] Yang L., Wang Y., Guo Y., Zhang W., Zhao Z. (2019). Robust working mechanism of water droplet-driven triboelectric nanogenerator: Triboelectric output versus dynamic motion of water droplet. Adv. Mater. Interfaces.

[60] Xu W., Zhou X., Hao C., Zheng H., Liu Y., Yan X., Yang Z., Leung M., Zeng X.C., Xu R.X. (2019). SLIPS-TENG: Robust triboelectric nanogenerator with optical and charge transparency using a slippery interface. Natl. Sci. Rev..

[61] Dong S., Xu F., Sheng Y., Guo Z., Pu X., Liu Y. (2020). Seamlessly knitted stretchable comfortable textile triboelectric nanogenerators for E-textile power sources. Nano Energy.

[62] Wu L., Xue P., Fang S., Gao M., Yan X., Jiang H., Liu Y., Wang H., Liu H., Cheng B. (2024). Boosting the output performance of triboelectric nanogenerators via surface engineering and structure designing. Mater. Horiz..

[63] Ulman A. (1996). Formation and structure of self-assembled monolayers. Chem. Rev..

[64] Hozumi A., Ushiyama K., Sugimura H., Takai O. (1999). Fluoroalkylsilane monolayers formed by chemical vapor surface modification on hydroxylated oxide surfaces. Langmuir.

[65] Song G., Kim Y., Yu S., Kim M.-O., Park S.-H., Cho S.M., Velusamy D.B., Cho S.H., Kim K.L., Kim J. (2015). Molecularly engineered surface triboelectric nanogenerator by self-assembled monolayers (METS). Chem. Mater..

[66] Kil Yun B., Kim J.W., Kim H.S., Jung K.W., Yi Y., Jeong M.-S., Ko J.-H., Jung J.H. (2015). Base-treated polydimethylsiloxane surfaces as enhanced triboelectric nanogenerators. Nano Energy.

[67] Byun K.-E., Cho Y., Seol M., Kim S., Kim S.-W., Shin H.-J., Park S., Hwang S.W. (2016). Control of triboelectrification by engineering surface dipole and surface electronic state. ACS Appl. Mater. Interfaces.

[68] Yao C., Yin X., Yu Y., Cai Z., Wang X. (2017). Chemically functionalized natural cellulose materials for effective triboelectric nanogenerator development. Adv. Funct. Mater..

[69] Lee J.H., Kim K.H., Choi M., Jeon J., Yoon H.J., Choi J., Lee Y.-S., Lee M., Wie J.J. (2019). Rational molecular design of polymeric materials toward efficient triboelectric energy harvesting. Nano Energy.

[70] Shanbedi M., Ardebili H., Karim A. (2023). Polymer-based triboelectric nanogenerators: Materials, characterization, and applications. Prog. Polym. Sci..

[71] Chen G., Dai Z., Li S., Huang Y., Xu Y., She J., Zhou B. (2021). Magnetically responsive film decorated with microcilia for robust and controllable manipulation of droplets. ACS Appl. Mater. Interfaces.

[72] Wang H., Zhang Z., Wang Z., Zhao J., Liang Y., Li X., Ren L. (2020). Improved dynamic stability of superomniphobic surfaces and droplet transport on slippery surfaces by dual-scale re-entrant structures. Chem. Eng. J..

[73] Dang Z.-M., Yuan J.-K., Zha J.-W., Zhou T., Li S.-T., Hu G.-H. (2012). Fundamentals, processes and applications of high-permittivity polymer–matrix composites. Prog. Mater. Sci..

[74] Zhang N. (2020). Study on Triboelectric Generator Based on Carbonyl Iron-PDMS Composite Film. Mater’s Dissertation.

[75] Wakino K., Okada T., Yoshida N., Tomono K. (1993). A New Equation for Predicting the Dielectric Constant of a Mixture. J. Am. Ceram. Soc..

[76] Sheng Y., Wang X., Qian J., Hu G., Huang Q., Lu H. (1989). Adjustment and Control of Dielectric Constant of Carbonyl Iron Powder. Mater. Technol. Spacecr..

[77] Ye C., Liu D., Gao Y., Liu F., Xu H., Jiang T., Wang Z.L. (2025). Electrostatic breakdown at liquid-solid-gas triple-phase interfaces owing to contact electrification. Matter.

